# Disruption of Glutamate Transport and Homeostasis by Acute Metabolic Stress

**DOI:** 10.3389/fncel.2021.637784

**Published:** 2021-02-02

**Authors:** Stefan Passlick, Christine R. Rose, Gabor C. Petzold, Christian Henneberger

**Affiliations:** ^1^Institute of Cellular Neurosciences, Medical Faculty, University of Bonn, Bonn, Germany; ^2^Institute of Neurobiology, Faculty of Mathematics and Natural Sciences, Heinrich Heine University Duesseldorf, Duesseldorf, Germany; ^3^German Center for Neurodegenerative Diseases (DZNE), Bonn, Germany; ^4^Division of Vascular Neurology, University Hospital Bonn, Bonn, Germany; ^5^Institute of Neurology, University College London, London, United Kingdom

**Keywords:** glutamate transport, EAAT, astrocyte, metabolic stress, ischemia, stroke, excitotoxicity

## Abstract

High-affinity, Na^+^-dependent glutamate transporters are the primary means by which synaptically released glutamate is removed from the extracellular space. They restrict the spread of glutamate from the synaptic cleft into the perisynaptic space and reduce its spillover to neighboring synapses. Thereby, glutamate uptake increases the spatial precision of synaptic communication. Its dysfunction and the entailing rise of the extracellular glutamate concentration accompanied by an increased spread of glutamate result in a loss of precision and in enhanced excitation, which can eventually lead to neuronal death *via* excitotoxicity. Efficient glutamate uptake depends on a negative resting membrane potential as well as on the transmembrane gradients of the co-transported ions (Na^+^, K^+^, and H^+^) and thus on the proper functioning of the Na^+^/K^+^-ATPase. Consequently, numerous studies have documented the impact of an energy shortage, as occurring for instance during an ischemic stroke, on glutamate clearance and homeostasis. The observations range from rapid changes in the transport activity to altered expression of glutamate transporters. Notably, while astrocytes account for the majority of glutamate uptake under physiological conditions, they may also become a source of extracellular glutamate elevation during metabolic stress. However, the mechanisms of the latter phenomenon are still under debate. Here, we review the recent literature addressing changes of glutamate uptake and homeostasis triggered by acute metabolic stress, i.e., on a timescale of seconds to minutes.

## Introduction

Excitatory synaptic transmission in the brain is mediated by the precisely timed and highly localized release of the neurotransmitter glutamate from presynaptic release sites into the synaptic cleft and the subsequent activation of postsynaptic receptors. After the initial dilution of glutamate in the synaptic cleft (Zheng et al., 2008; Scimemi and Beato, 2009), the fast clearance of glutamate from the extracellular space (ECS) restricts its spread. Thereby, it limits the activation of extrasynaptic receptors and the amount of glutamate “spilling over” onto nearby neurons. High-affinity glutamate transporters located on the fine perisynaptic processes of astrocytes (PAPs) are believed to mediate most of the glutamate clearance in the brain under physiological conditions (Danbolt, 2001; Rose et al., 2017). Multiple studies have demonstrated the role of glutamate uptake for synaptic transmission and plasticity (for review see Tzingounis and Wadiche, 2007; Valtcheva and Venance, 2019). However, its relevance becomes particularly apparent when it is impaired (Rose et al., 2017; Pajarillo et al., 2019). The uptake of glutamate against its steep concentration gradient is mainly powered by the transmembrane driving force for Na^+^ at a negative membrane potential, both of which are maintained by the Na^+^/K^+^-ATPase. During conditions of metabolic stress such as ischemic stroke, the lack of ATP leads to a breakdown of ionic gradients and cellular depolarization, reducing the driving force for glutamate uptake. The resulting increased lifetime of glutamate in the ECS leads to increased activation of synaptic and extrasynaptic glutamate receptors and receptors on nearby neurons, which provokes further excitation and intracellular Ca^2+^ elevations, driving a vicious circle eventually culminating in irreversible excitotoxic cell death (Dirnagl et al., 1999; Grewer et al., 2008).

In an ischemic stroke, two characteristic zones can be distinguished (Rossi et al., 2007). The ischemic core is in the direct vicinity of the clogged blood vessel, where energy supply is almost completely lost and irreversible cell death occurs within minutes. The tissue surrounding the core, called the penumbra, suffers from reduced perfusion and thus lower energy levels but recovery is still possible. However, peri-infarct depolarizations (PIDs), which are waves of spreading depression that originate in the core and propagate through the penumbra into the healthy tissue, impose an additional metabolic burden on the tissue (Dirnagl et al., 1999; Rossi et al., 2007; Dreier, 2011).

Disentangling the sequence of events and identifying the relevant cellular mechanisms during this acute form of metabolic stress is vital for developing novel treatments. Indeed, multiple studies have shown that glutamate homeostasis is perturbed and, in many ways, responsible for ischemic cell death. However, some findings are still controversial (Rossi et al., 2007; Rose et al., 2017; Zhang et al., 2019; Belov Kirdajova et al., 2020) and there are multiple reasons for that. These include the complexity of the pathological condition itself, the large number of potentially relevant cellular mechanisms, and the variety of experimental conditions across different studies. In this mini-review, we discuss the impact of metabolic stress on glutamate homeostasis in the cortex, focusing on mechanisms that are activated during transient (seconds to minutes) episodes of metabolic stress, as is for instance the case in the penumbra after a stroke or during transient ischemic attacks.

## Transporter Availability and Location

### Expression of Glutamate Transporters

Mammalian cells express five different subtypes of excitatory amino acid transporters (EAAT1–5; Danbolt, 2001). In the hippocampus and neocortex, EAAT1 (= glutamate/aspartate transporter, GLAST) and EAAT2 (= glutamate transporter-1, GLT-1) are the predominantly expressed subtypes. However, the relative expression and functional significance for the uptake of synaptically released glutamate can vary significantly even within subregions of the neocortex (Romanos et al., 2019). EAAT1 and EAAT2 are considered to be mainly astrocytic while EAAT3 (= excitatory amino acid carrier 1, EAAC1) and EAAT4 are predominantly found in neurons (Danbolt, 2001; Massie et al., 2008; Holmseth et al., 2012; Rose et al., 2017). In the adult hippocampus, for instance, it was shown that ~10% of EAAT2 is expressed by neurons where it localizes mainly to axon terminals (Danbolt, 2001; Furness et al., 2008) while neuronal EAAT3 on the other hand is located on the postsynaptic membrane (Holmseth et al., 2012). While the density of glutamate transporters is significantly higher on astrocytes compared to neurons (for the hippocampus see for instance Holmseth et al., 2012), all subtypes are strategically located close to the synaptic glutamate release site.

Changes in glutamate transporter expression can occur on multiple levels, including transcriptional control, epigenetic DNA modifications, and regulation of translation. Furthermore, post-translational modifications such as phosphorylation, nitrosylation, and ubiquitination determine the availability of functional glutamate transporters (Pajarillo et al., 2019). For instance, EAAT2 is subject to nitrosylation, which was shown to influence uptake efficiency (Raju et al., 2015). In the case of ischemia, the extent of expression changes depends on the stage and/or severity of metabolic stress although results in the literature are heterogeneous (Zhang et al., 2019). While some studies indicate that expression of EAAT1 and/or EAAT2 is increased in the early stages of an ischemic event and decreased in later stages (Arranz et al., 2010; Liu et al., 2012; Girbovan and Plamondon, 2015), others demonstrated the opposite (Torp et al., 1995; Rao et al., 2001a; Fang et al., 2014). Intriguingly, it was also shown more recently that EAAT1 and EAAT2 mRNAs are found in astrocyte processes along with the machinery for local translation and that the distribution of mRNAs was altered after fear conditioning (Sakers et al., 2017; Mazaré et al., 2020). It is therefore tempting to speculate that changes in the translation of glutamate transporters might also occur locally in response to acute metabolic stress. However, such changes of transporter expression are likely to play a role over longer time scales than the seconds to minutes of interest here.

### Localization of Glutamate Transporters

The availability of functional glutamate transporters depends, apart from transcription and translation, on the regulation of glutamate transporter insertion and internalization as well as lateral diffusion in the cell membrane. Insertion and internalization were shown to change in response to glutamate application (Duan et al., 1999) and are likely to be regulated by Ca^2+^ dynamics (Rose et al., 2017). Substances promoting retention of glutamate transporters in the membrane were demonstrated to have neuroprotective effects, similar to increasing expression (Martínez-Villarreal et al., 2012; Li et al., 2015). Single-molecule tracking experiments revealed that, once inside the membrane, glutamate transporters are quite mobile, their diffusion is modulated by neuronal activity and they become more stable near synapses (Murphy-Royal et al., 2015). Likewise, glutamate application was shown to affect the formation and stability of EAAT2 clusters inside the astrocyte membrane (Al Awabdh et al., 2016) while blocking synaptic activity reduced the density and perisynaptic localization of EAAT2 clusters in developing astrocytes (Benediktsson et al., 2012). Since inhibiting transporter mobility directly affected synaptic transmission (Murphy-Royal et al., 2015), there is a dynamic and plastic interplay between synaptic glutamate release and local transporter availability. Furthermore, elevated glutamate levels increased ubiquitination of EAAT2 leading to its internalization in HEK293 cells (Ibáñez et al., 2016). For these reasons, it is plausible that PIDs, associated with large extracellular glutamate and intracellular Ca^2+^ transients (Rakers and Petzold, 2017), may affect transporter membrane diffusion, insertion, and internalization. However, little is known on how metabolic stress precisely affects glutamate transporter trafficking. It, therefore, remains to be established if such processes play a role in, for instance, the initial stages of an ischemic stroke or during PIDs.

In addition to the density of glutamate transporters in the membrane, the physical distance between the glutamate release site and the transporters directly influences the lifetime and spread of glutamate in the ECS. Neuronal membranes facing the synaptic cleft itself contain few transporters (Furness et al., 2008). However, after escaping the synaptic cleft, the radius of action of glutamate is determined by diffusion and the hindrance thereof either by physical barriers or by binding and uptake by transporters (Zheng et al., 2008; Scimemi and Beato, 2009). Hence, glutamate transporters located close to the synaptic cleft limit the diffusion of glutamate to extrasynaptic sites, where high-affinity *N*-methyl-D-aspartate receptors (NMDARs) and metabotropic glutamate receptors (mGluRs) are localized, and prevent the spillover to neighboring neurons (Scimemi et al., 2004; Zheng et al., 2008; Henneberger et al., 2020; Herde et al., 2020).

Under physiological conditions, glutamate uptake is predominantly mediated by astrocytic glutamate transporters (Danbolt, 2001; Rose et al., 2017). For this reason, the perisynaptic geometry and abundance of PAPs and their role in glutamate uptake has been intensely studied. A reduction of astroglial coverage of synapses during lactation in the supraoptic nucleus, for example, was shown to increase glutamate spread leading to changes in presynaptic transmitter release (Oliet et al., 2001). In contrast, invasion of the synaptic cleft by astrocytic processes in Connexin 30 knockout mice increased the control of synaptic transmission by glutamate uptake (Pannasch et al., 2014). Along these lines, we recently demonstrated that spine size negatively correlates with the local efficacy of glutamate uptake in the hippocampus (Herde et al., 2020). Another study suggested that rearrangements of the astrocytic cytoskeleton induced changes in glutamate uptake which were linked to altered activity of the Na^+^/K^+^-ATPase (Sheean et al., 2013). Together, these results highlight that even small changes in the submicron structure of astrocytic processes can have significant consequences for the time course of extracellular glutamate transients and thus on the activation of extrasynaptic glutamate receptors and neighboring neurons. Since changes in cellular morphology as a consequence of ischemia and metabolic stress have been described for different cell types including astrocytes (Risher et al., 2009; Anderova et al., 2011), it is safe to assume that these will directly affect extracellular glutamate dynamics after the onset of metabolic stress.

Notably, we and others have also shown that astrocytic processes are mobile (Haber et al., 2006; Bernardinelli et al., 2014) and withdraw from synapses after induction of long-term potentiation (LTP) of synaptic transmission, which increases glutamate spread and spillover enhancing NMDAR activation (Henneberger et al., 2020). Interestingly, brief episodes of metabolic stress were shown to induce LTP too, a phenomenon known as ischemic LTP (Lenz et al., 2015) that also depends on NMDARs (Maggio et al., 2015). This raises the question if metabolic stress induces a similar withdrawal of astrocytic processes or whether swelling of astrocytic processes (Risher et al., 2009) is more prominent, and how either displacement of glutamate transporters changes extracellular glutamate dynamics in the perisynaptic space.

## Impairment of Transporter Function

### Reduced Uptake and Reversal-Ion Homeostasis and the Direction of Glutamate Transport

When compared to the sub-micromolar extracellular glutamate concentration (Herman and Jahr, 2007), the intracellular glutamate concentration in neurons and astrocytes is much higher with estimates ranging from high micromolar to low millimolar (Nedergaard et al., 2002; Rossi et al., 2007). Glutamate uptake against this steep concentration gradient is achieved by the co-transport of three Na^+^ and one H^+^ and the export of one K^+^ and is accompanied by an uncoupled anion flux (Danbolt, 2001; Fahlke and Nilius, 2016). Therefore, glutamate transport is very sensitive to changes of the Na^+^ gradient.

The Na^+^ gradient across the membrane is largely maintained by the Na^+^/K^+^-ATPase and therefore highly dependent on energy in the form of ATP. During metabolic stress, reduced levels of ATP lead to malfunction of the Na^+^/K^+^-ATPase and cellular depolarization, a degradation of ionic gradients, and also extracellular glutamate accumulation (Rossi et al., 2007). Cellular depolarization and the degradation of ion gradients both directly reduce the driving force for Na^+^ and, thereby, glutamate transport. Also, due to the high K^+^ membrane conductance of astrocytes, extracellular rises in K^+^ during metabolic stress strongly depolarize these cells, which further reduces the driving force for glutamate uptake. Furthermore, extracellular accumulation of glutamate likely accelerates the decay of ionic gradients in cells with a large contribution to glutamate uptake such as astrocytes, because of the ion transport associated with it. During extreme energy failure, e.g., in the core region of an ischemic stroke, this may lead to a reversal of glutamate uptake, i.e., to the export of glutamate from the cytosol to the ECS (Rossi et al., 2000; Grewer et al., 2008). Whether or not such a reversal occurs depends on how strongly the ionic gradients and membrane potential are degraded. Modeling studies of glutamate transport predict that determining this tipping point requires quantitative information about the intra- and extracellular concentrations of glutamate, Na^+^, K^+^, and H^+^ in the course of metabolic stress (Bergles et al., 2002; Rossi et al., 2007; Grewer et al., 2008), which is currently not available.

**Figure 1:**
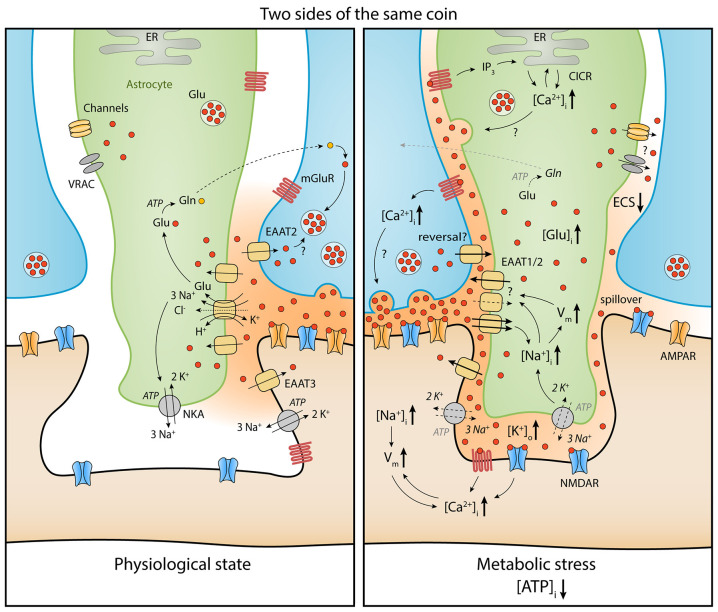
Comparison of extracellular glutamate (red) homeostasis under physiological conditions (left) and during metabolic stress (right) at a synapse (presynaptic bouton in blue, postsynaptic dendritic spine in brown, perisynaptic astrocyte process in green). A moderate reduction of ATP in astrocytes and neurons leads to depolarization of the membrane potential (V_m_) and intracellular increases of Na^+^ and Ca^2+^ as well as extracellular increases of K^+^. As a consequence, the driving force for glutamate uptake is diminished and cell swelling reduces the extracellular space (ECS) fraction, which increases extracellular glutamate levels (see text). This leads to stronger activation of glutamate receptors on neurons (e.g., *N*-methyl-D-aspartate receptors, NMDARs) and astrocytes (e.g., metabotropic glutamate receptors, mGluRs) and a further increase in intracellular Ca^2+^ and Na^+^ concentrations and depolarization. In astrocytes, glutamate release through volume-regulated anion channels (VRACs), as well as Ca^2+^-dependent mechanisms, can amplify extracellular glutamate increases during acute metabolic stress. Open questions (?) regarding glutamate homeostasis are related to the quantitative role of glutamate transporter dysfunction, the relationship between acute metabolic stress and changes of presynaptic release, the role of active astrocytic signaling, and the geometrical rearrangement of astrocytes and neurons during metabolic stress and its impact on extracellular glutamate spread. Abbreviations: AMPAR, *α*-amino-3-hydroxy-5-methyl-4-isoxazolepropionic acid receptor; CICR, Ca^2+^-induced Ca^2+^ release; EAAT, excitatory amino acid transporter; ECS, extracellular space; ER, endoplasmatic reticulum; Gln, Glutamine; Glu, Glutamate; IP_3_, inositol triphosphate; mGluR, metabotropic glutamate receptor; NKA, Na^+^/K^+^-ATPase; NMDAR, N-methyl-D-aspartate receptor; VRAC, volume-regulated anion channel. Note: schematic distribution of glutamate molecules not directly representative of their concentration.

Importantly, an impairment of glutamate uptake is likely sufficient to facilitate the failure of extracellular glutamate homeostasis. Indeed, even moderate depolarization of the astrocytic membrane potential (Stephan et al., 2012) and/or increases of the Na^+^ concentration (Kelly et al., 2009) were shown to reduce the uptake capacity significantly (Felix et al., 2020). We recently demonstrated that even transient episodes of metabolic stress during chemical ischemia in acute brain slices as well as during PIDs *in vivo* induce significant increases in the neuronal as well as astrocytic Na^+^ concentration (Gerkau et al., 2018). Thus, acute and/or transient metabolic stress can impair glutamate uptake substantially. This will promote local glutamate accumulation and glutamate spread into the tissue (Figure 1) because inhibition of glutamate uptake increases the lifetime of glutamate in the ECS and its spread (Henneberger et al., 2020; Herde et al., 2020). However, glutamate uptake is not the only mechanism that determines extracellular glutamate concentrations and their diffusion. It is for instance well-known that ischemia and metabolic stress can induce cell swelling and a decrease of the ECS (Syková and Nicholson, 2008; Risher et al., 2009; Anderova et al., 2011). The same amount of glutamate released into a smaller ECS would intuitively lead to higher extracellular glutamate concentrations. At the same time, the tortuosity of the ECS can be increased by ischemia (Syková and Nicholson, 2008), which would on its own reduce the diffusion of glutamate in the ECS. Therefore, it will be interesting to test what the net effect of metabolic stress on glutamate spread in the ECS is.

Interestingly, regional variations in glutamate uptake mechanisms could lead to differential susceptibility to metabolic stress across brain regions. For instance, the differentially expressed glutamate transporters EAAT1 and EAAT2 were shown to differ in their uptake kinetics with EAAT2 being more effective (Arriza et al., 1994). Furthermore, it was shown that glutamate uptake in the cortex was slowed by increased presynaptic activity (Armbruster et al., 2016) while no such adaption was found in the hippocampus (Diamond and Jahr, 2000; Diamond, 2005). Similarly, employing the glutamate sensor iGluSnFR, other studies have found that glutamate clearance is faster in the hippocampus compared to the cortex (Pinky et al., 2018) and that its activity-dependence differs between somatosensory and frontal cortex (Romanos et al., 2019). Although the mechanisms underlying this activity-dependence are not fully established, it seems likely that they are engaged by the widespread glutamate increases accompanying PIDs (Fabricius et al., 1993; Rakers and Petzold, 2017) and, in fact, also contribute to the latter.

Once taken up by astrocytes, the enzyme glutamine synthetase (GS) converts glutamate to glutamine. The non-toxic glutamine is then shuttled back to neurons where it is converted to glutamate or GABA (Schousboe et al., 2014). The ATP-dependent conversion by GS is also inhibited during metabolic stress likely inducing glutamate accumulation in astrocytes further reducing the driving force for glutamate uptake. Consequently, it was shown that inhibiting GS increased NMDAR currents in cortical neurons indicating that accumulation of glutamate in astrocytes reduced the uptake capacity promoting glutamate spread (Trabelsi et al., 2017). Similarly, a modeling study found a direct relationship between intracellular glutamate concentration and glutamate uptake capacity (Flanagan et al., 2018).

In summary, although many links are still missing, present data indicate that acute metabolic stress affects a wide range of cellular processes that directly interfere with glutamate clearance *via* transporters, which in turn can affect the accumulation and spread of glutamate in the ECS (Figure 1).

### Neuronal vs. Glial Glutamate Transporters

The precise differential contribution of astrocytic and neuronal glutamate transporters to glutamate clearance during physiological but also pathophysiological conditions remains a matter of debate. The commonly accepted notion is that astrocytes take up >90% of the synaptically released glutamate (Danbolt, 2001; Diamond, 2005; Rose et al., 2017). This is supported by genetic ablation studies which found that an astrocyte-specific knockout of EAAT2 showed by far the most severe phenotype of all transporter knockouts including strong seizure activity leading to neonatal death (Petr et al., 2015). However, constitutive as well as neuron-specific knockout experiments indicated that neuronal EAAT2 is particularly important for glutamate uptake into synaptosomes (Furness et al., 2008; Petr et al., 2015). It should also be noted that the knockout approach depicts the most drastic model to study protein function and compensatory effects need to be considered. Also, the relative impact of neuronal glutamate uptake might increase during acute metabolic stress when astrocytic glutamate uptake is compromised though not entirely lost.

Still, most studies identified glial glutamate transporters as the main source of glutamate *via* reverse transport during severe ischemia whereas increased glutamate release from neurons is mainly *via* vesicular release (Jabaudon et al., 2000; Andrade and Rossi, 2010). Other reports, however, suggested that the reversal of neuronal glutamate transporters is responsible for glutamate release during early phases of ischemia (Rossi et al., 2000; Hamann et al., 2002). This was supported by the observation that myelin damage in the corpus callosum during ischemic conditions is almost exclusively caused by axonal glutamate release while reverse transport by astrocytes did not contribute significantly (Doyle et al., 2018). On the other hand, selective knockdown of the mainly astrocytic glutamate transporter EAAT2 increased infarct volume in an *in vivo* model of stroke while knockdown of the primarily neuronal EAAT3 had no such effect (Rao et al., 2001b). Furthermore, it was recently demonstrated that brain slices from mice with astrocyte-specific EAAT2 deletion were more susceptible to spreading depression compared to EAAT1 or EAAT3 knockout mice indicating a stronger influence of glial compared to neuronal glutamate transporters (Aizawa et al., 2020).

Although the exact contributions of neuronal vs. glial glutamate transporters during metabolic stress are still under debate, it is quite likely that both are dysregulated at some stage and thus both do contribute to the failure of extracellular glutamate homeostasis. The degree to which either cell type plays a role will not only depend on its physiological contribution to glutamate uptake but also on how metabolic stress impairs concentration gradients and the membrane potential in a given cell type (Rossi et al., 2007).

### Astrocytic Glutamate Release Beyond Reverse Transport

While the vesicular and non-vesicular routes of glutamate release from neurons are established (see above for glutamate transporters), mechanisms of astrocytic glutamate release are still debated. In addition to reverse transport, several studies indicate that astrocytes are capable of regulated, and in many cases Ca^2+^-dependent, vesicular as well as channel-mediated glutamate release (Bohmbach et al., 2018; Mahmoud et al., 2019). We recently showed that intracellular Na^+^ increases during acute metabolic stress induce Ca^2+^ elevations *via* reversal of the Na^+^/Ca^2+^-exchanger in neurons and astrocytes (Gerkau et al., 2018). Furthermore, we demonstrated that preventing inositol triphosphate receptor type 2-mediated release of Ca^2+^ from intracellular stores reduced the PID-induced Ca^2+^ increases in astrocytes and, at the same time, the duration of extracellular glutamate transients *in vivo*, leading to a secondary neuroprotective decrease of Ca^2+^ elevations in neurons (Rakers and Petzold, 2017). This suggests that astrocytic Ca^2+^ signals during PIDs could trigger astrocytic glutamate release, although the exact chain of events remains to be uncovered. Another relevant mechanism is astrocytic release *via* volume-regulated anion channels (VRACs), which are Ca^2+^-independent (Mahmoud et al., 2019; Yang et al., 2019). Importantly, VRACs were implicated in glutamate release in the penumbra while reversal of transport was suggested to be more important in the core (Feustel et al., 2004). Also, the VRAC Swell1 was recently shown to mediate ischemia-induced glutamate release from astrocytes with Swell1 knockout mice exhibiting significantly smaller infarct volumes (Yang et al., 2019). Together, these studies indicate that astrocytes can drive glutamate elevations during metabolic stress, ischemia, and stroke by mechanisms independent from high-affinity glutamate transporters.

## Conclusion

Accumulating data indicate that metabolic stress acutely impairs glutamate uptake promoting glutamate accumulation and spread further aggravating excitotoxicity. Glutamate transporter dysfunction scales with the severity of metabolic stress ranging from subtle changes of uptake efficiency to a reversal of uptake. A variety of factors can potentially contribute, including lateral diffusion, localization, and abundance of glutamate transporters, their biophysical properties, and their driving forces. Given this complexity, it is no surprise that disentangling the sequence of events triggered by acute metabolic stress has remained challenging. Novel tools such as optical glutamate sensors (Marvin et al., 2013; Helassa et al., 2018) have more recently allowed researchers to gain new insights into extracellular glutamate dynamics and the role of glutamate transporters (Armbruster et al., 2016, 2020; Romanos et al., 2019; Henneberger et al., 2020; Herde et al., 2020). They also promise to provide new insights into unresolved questions regarding altered glutamate transport in metabolic stress (Rakers and Petzold, 2017). Regarding glutamate excitotoxicity, it will also be important to reveal if metabolic stress also perturbs NMDAR co-agonist supply and transport (see Henneberger et al., 2013), which could amplify the damage caused by impaired glutamate homeostasis.

## Author Contributions

All authors contributed to the conception and writing of this review and approved the submitted and final published version.

## Conflict of Interest

The authors declare that the research was conducted in the absence of any commercial or financial relationships that could be construed as a potential conflict of interest.
